# Pre-Transplant Endothelial Activation and Stress Index (EASIX) and Albumin-Modified mEASIX (mEASIX_alb) Predict Survival in Multiple Myeloma Patients Undergoing Autologous Stem Cell Transplantation

**DOI:** 10.3390/medicina62081572

**Published:** 2026-08-17

**Authors:** Tuba Güllü Koca, Nizameddin Koca, Fazıl Çağrı Hunutlu, Sinem Çubukçu, Rümeysa Yılmaz, Tuba Ersal, Vildan Gürsoy, Ibrahim Ethem Pınar, Vildan Özkocaman, Fahir Özkalemkaş

**Affiliations:** 1 Department of Hematology, Bursa Şehir Training & Research Hospital, University of Health Sciences, 16110 Bursa, Türkiye; 2 Department of Internal Medicine, Bursa Şehir Training & Research Hospital, University of Health Sciences, 16110 Bursa, Türkiye; 3 Department of Hematology, Ali Osman Sonmez Oncology Hospital, 16040 Bursa, Türkiye; 4 Department of Hematology, Bursa Uludağ University Hospital, Bursa Uludağ University, 16059 Bursa, Türkiye

**Keywords:** multiple myeloma, autologous stem cell transplantation, EASIX, endothelial stress, prognostic nutritional index, PNI, survival

## Abstract

*Background and Objectives*: Endothelial dysfunction is increasingly recognized as a key determinant of transplant outcomes. The Endothelial Activation and Stress Index (EASIX), originally validated in allogeneic stem cell transplantation, integrates markers of endothelial stress into a single composite score. Its prognostic role in autologous stem cell transplantation (ASCT) for multiple myeloma (MM) remains undefined. We evaluated the pre-transplant prognostic value of EASIX, an albumin-modified formulation (mEASIX_alb), and nutritional indices (Prognostic Nutritional Index [PNI] and Controlling Nutritional Status [CONUT] score) in a large MM-ASCT cohort. *Materials and Methods*: We retrospectively analyzed 274 MM patients who underwent ASCT at a single institution. The pre-ASCT EASIX (LDH × Creatinine/Platelets), mEASIX_alb (LDH × Creatinine/[Platelets × Albumin]), PNI (10 × Albumin [g/dL] + 0.005 × Lymphocyte count), and CONUT score were calculated from laboratory values obtained on day 3. Optimal binary cutoffs were determined by maximally selected rank statistics. Overall survival (OS) and progression-free survival (PFS) were analyzed with Cox models in which EASIX and mEASIX_alb were entered as continuous, log-transformed variables; binary cutoffs were used only for descriptive Kaplan–Meier illustration. Internal model validity was assessed by bootstrap resampling (B = 1000). *Results*: Over a median follow-up of 81.3 months, 150 patients (54.7%) died, and 207 (75.5%) experienced disease progression or death. Using illustrative cutoffs (EASIX > 0.471, mEASIX_alb > 0.208, PNI < 52.29), higher EASIX was associated with shorter OS (66.7 vs. 108.1 months; *p* = 0.012) and PFS (27.4 vs. 42.7 months; *p* = 0.018). In multivariable analysis, log-transformed EASIX (HR 1.40, 95% CI 1.10–1.77; *p* = 0.006) and mEASIX_alb (HR 1.38, 95% CI 1.09–1.73; *p* = 0.007) independently predicted OS after adjustment for baseline covariates (ISS stage, pre-transplant response, and age). EASIX remained independently prognostic in a day-100 landmark analysis (HR 1.35; *p* = 0.023). Critically, the PNI, prognostic for OS in univariate analysis (HR 0.973; *p* = 0.038), did not provide independent incremental prognostic value beyond the clinical model when EASIX was added (HR 0.99; *p* = 0.526); this does not establish shared information between EASIX and PNI. *Conclusions*: Pre-transplant EASIX and mEASIX_alb are independent prognostic biomarkers in MM-ASCT. The PNI did not provide independent incremental prognostic value beyond the clinical model. These scores may inform pre-transplant risk stratification, although the illustrative cutoffs require external validation before clinical use. EASIX retained prognostic significance across transplant eras and in a cytogenetically characterized subset, supporting its era-independent and cytogenetics-independent utility.

## 1. Introduction

Multiple myeloma (MM) is the second most common hematologic malignancy, accounting for approximately 10% of all blood cancers [1]. Despite the introduction of novel agents, including proteasome inhibitors, immunomodulatory drugs, and anti-CD38 monoclonal antibodies, MM remains incurable in the majority of patients. High-dose melphalan followed by autologous stem cell transplantation (ASCT) remains the standard consolidation strategy for transplant-eligible MM patients, conferring meaningful improvements in progression-free and overall survival [2,3].

Despite well-established disease-related prognostic markers, such as ISS stage, cytogenetic risk, and depth of pre-transplant response, there remains significant heterogeneity in post-ASCT outcomes that these factors do not fully explain. This underscores the need for additional pre-transplant biomarkers that capture the patient’s biological reserve and susceptibility to transplant-related stress. In particular, markers of endothelial health and nutritional status have emerged as promising candidates for risk stratification in hematologic malignancies [4,5].

The Endothelial Activation and Stress Index (EASIX) was originally developed to quantify endothelial stress in allogeneic SCT, incorporating lactate dehydrogenase (LDH), serum creatinine, and platelet count, reflecting endothelial injury (LDH), organ stress (creatinine), and consumptive endotheliopathy (thrombocytopenia) [6]. Calculated as EASIX = LDH (U/L) × Creatinine (mg/dL)/Platelet count (×10^9^/L), this index has been validated as a predictor of mortality and transplant-related complications in multiple allogeneic SCT cohorts [6,7,8,9]. Its potential utility in the ASCT setting, where conditioning-related endothelial injury is milder, and graft-versus-host disease biology does not apply, is biologically plausible but incompletely explored.

Nutritional assessment offers a complementary biological window. The Prognostic Nutritional Index (PNI), combining serum albumin and lymphocyte count, and the Controlling Nutritional Status (CONUT) score, integrating albumin, cholesterol, and lymphocyte count, have demonstrated prognostic value across solid and hematologic malignancies [10,11]. Albumin, a component of both indices, is also an established marker of systemic inflammation and endothelial integrity, suggesting a mechanistic link between endothelial and nutritional stress pathways.

We hypothesized that: (1) pre-ASCT EASIX and its albumin-modified derivative (mEASIX_alb) would independently predict survival in MM patients; (2) nutritional indices (PNI, CONUT) would show complementary prognostic value; and (3) given their shared association with albumin-related and inflammatory pathways, the nutritional indices might not remain independently prognostic when analyzed jointly with EASIX. To test these hypotheses, we conducted a retrospective analysis of 274 MM patients who underwent ASCT at a single center.

## 2. Materials and Methods

### 2.1. Study Design and Patient Population

This retrospective cohort study included consecutive MM patients who underwent ASCT at Bursa Uludağ University Hospital, Bursa, Türkiye, between January 2010 and May 2023. The inclusion criteria were: (1) confirmed MM diagnosis per IMWG criteria; (2) first ASCT with available pre-transplant laboratory data on day 3; and (3) minimum post-transplant follow-up of 30 days. Patients undergoing a second or tandem ASCT were represented by their first transplant only. The study was approved by the Bursa Uludag University Clinical Research Ethics Committee (Approval No: 2023-16/8), and the requirement for individual informed consent was waived due to the retrospective design.

### 2.2. Index Timepoint and Laboratory Assessment

All biomarkers were derived from routine laboratory values collected on day 3 relative to ASCT (the day before conditioning initiation), representing the pre-conditioning baseline. The variables included: lactate dehydrogenase (LDH, U/L), serum creatinine (mg/dL), platelet count (×10^9^/L), serum albumin (g/dL), lymphocyte count (/mm^3^), and total cholesterol (mg/dL).

### 2.3. Score Calculations

EASIX = LDH (U/L) × Creatinine (mg/dL)/Platelet count (×10^9^/L) [6].

mEASIX_alb = LDH × Creatinine/(Platelet count [×10^9^/L] × Albumin [g/dL]).

A CRP-modified formulation (mEASIX_crp = LDH × CRP/Platelet count) was also evaluated; CRP values below the detection limit were imputed by the LOD/2 method.

PNI (Prognostic Nutritional Index) = 10 × Albumin (g/dL) + 0.005 × Lymphocyte count (/mm^3^) [10].

The CONUT (Controlling Nutritional Status) score was computed per published criteria from albumin (0–6 points), total cholesterol (0–3 points), and lymphocyte count (0–3 points; total 0–12) [12].

### 2.4. Clinical Variables

ISS stage (I/II/III) was used as an ordinal variable. The pre-transplant response was classified by IMWG criteria [13] as a complete response (CR), very good partial response (VGPR), or partial response (PR). Conditioning was classified as melphalan 200 mg/m^2^ (MEL200) or as reduced-intensity conditioning. Lenalidomide maintenance was recorded as yes/no. Cytogenetic risk by FISH (t[4;14], t[14;16], del[17p]) was available in only 32% of patients and was excluded from primary multivariable models. Neutrophil engraftment was defined as ANC ≥ 0.5 × 10^9^/L for three consecutive days; platelet engraftment as PLT ≥ 20 × 10^9^/L for three consecutive days without transfusion support.

**Endpoints**

Overall survival (OS) was defined as the time from ASCT to death from any cause. Progression-free survival (PFS) was the time from ASCT to disease progression per IMWG criteria, death, or last follow-up.

### 2.5. Statistical Analysis

Optimal binary cutoffs were identified by maximally selected rank statistics (maxstat R package) [14]. Kaplan–Meier curves were compared by the log-rank test. Three OS multivariable Cox models were specified: Model 1 (EASIX + clinical covariates), Model 2 (mEASIX_alb + clinical covariates), and Model 3 (EASIX + PNI + clinical covariates, to test whether the PNI retains an independent value after accounting for EASIX). Two parallel PFS models were specified. The baseline (pre-ASCT) covariates selected a priori for the primary models were ISS stage, pre-transplant response, and age. Lenalidomide maintenance, a post-transplant exposure, was not included in the primary models: because it can only begin in patients who remain progression-free after transplant, modeling it as a baseline covariate would introduce immortal-time bias. Its association with outcome was instead examined in a day-100 landmark analysis restricted to patients event-free and under follow-up at day 100, with survival measured from the landmark and adjustment for the same baseline covariates as the primary models. Because individual maintenance start dates were not recorded, maintenance was classified by receipt during follow-up rather than by status precisely at day 100; residual immortal-time bias for the maintenance term therefore cannot be fully excluded, and the landmark analysis is interpreted primarily as a robustness check for EASIX. The proportional-hazards assumption was tested for all models using scaled Schoenfeld residuals. EASIX and mEASIX_alb were modeled as continuous, log-transformed variables, and the binary cutoffs were retained for descriptive Kaplan–Meier illustration only. A combined risk score was constructed (EASIX > 0.471 [1 point] + PNI < 52.29 [1 point]; range 0–2). Engraftment differences were compared by the Wilcoxon rank-sum test. Spearman correlations were computed for continuous score relationships. Two-tailed *p* < 0.05 was significant. For analyses, R version 4.6.0 was used; for figures, ggplot2, survminer, and cowplot were used. For internal model validation, bootstrap resampling (B = 1000, seed = 2025) was used, assessing both Harrell’s C-index and cutoff stability (rms package). A sensitivity analysis evaluated continuous EASIX in patients with available FISH data, with and without adjustment for high-risk cytogenetics. Era-independence was assessed by adding transplant year (pre-2018 vs. post-2018) to primary multivariable models.

## 3. Results

### 3.1. Patient Characteristics

Between January 2010 and May 2023, 274 MM patients who underwent ASCT met the inclusion criteria (Table 1). The cohort comprised 149 women (54.4%) and 125 men (45.6%), with a median age at transplant of 57.8 years (IQR 51.4–63.2). The most common myeloma subtype was IgG (n = 151, 55.1%), followed by light-chain disease (n = 60, 21.9%) and IgA (n = 47, 17.2%). The ISS stage at diagnosis was stage I in 42 (15.4%), stage II in 89 (32.6%), and stage III in 141 (51.5%) patients. The pre-transplant response was a CR in 156 (57.1%), VGPR in 84 (30.7%), and PR in 33 (12.1%) patients. Bortezomib–dexamethasone–cyclophosphamide (VCD)-based induction was administered to 187 patients (68.2%), VD to 61 (22.3%), and other/multiple regimens to 26 (9.5%). MEL200 conditioning was used in 254 patients (92.7%). Lenalidomide maintenance was prescribed in 184 patients (67.2%). Median neutrophil engraftment occurred at day + 11 (IQR 11–12) and platelet engraftment at day + 13 (IQR 11–15). Over a median follow-up of 81.3 months (reverse Kaplan–Meier method), 150 patients (54.7%) had died, and 207 (75.5%) had experienced disease progression or death. The median OS was 71.5 months (95% CI 62.5–89.0) and the median PFS was 29.0 months (95% CI 23.9–33.9).

**Pre-ASCT Score Distributions**

The median LDH was 191 U/L (IQR 160.5–227), creatinine 0.77 mg/dL (IQR 0.67–1.00), and platelet count 228 × 10^9^/L (IQR 189–278). Albumin was available in all 274 patients (median 4.1 g/dL). EASIX was calculable in 240 patients (87.6%; 34 excluded due to missing LDH), with a median of 0.699 (IQR 0.490–1.049). The median mEASIX_alb was 0.176 (IQR 0.119–0.262) and the median PNI was 49.0 (IQR 45.5–52.3).

### 3.2. Optimal Cutoff Determination

The optimal OS-based cutoff for EASIX was 0.471, yielding 51 patients (21.3%) in the low-risk group and 189 (78.8%) in the high-risk group (among n = 240 with available data). The optimal cutoff for mEASIX_alb was 0.208, and for PNI it was 52.29 (PNI ≥ 52.29 = favorable; n = 68, 24.9%).

### 3.3. Kaplan–Meier Survival Analysis

**Figure 1:**
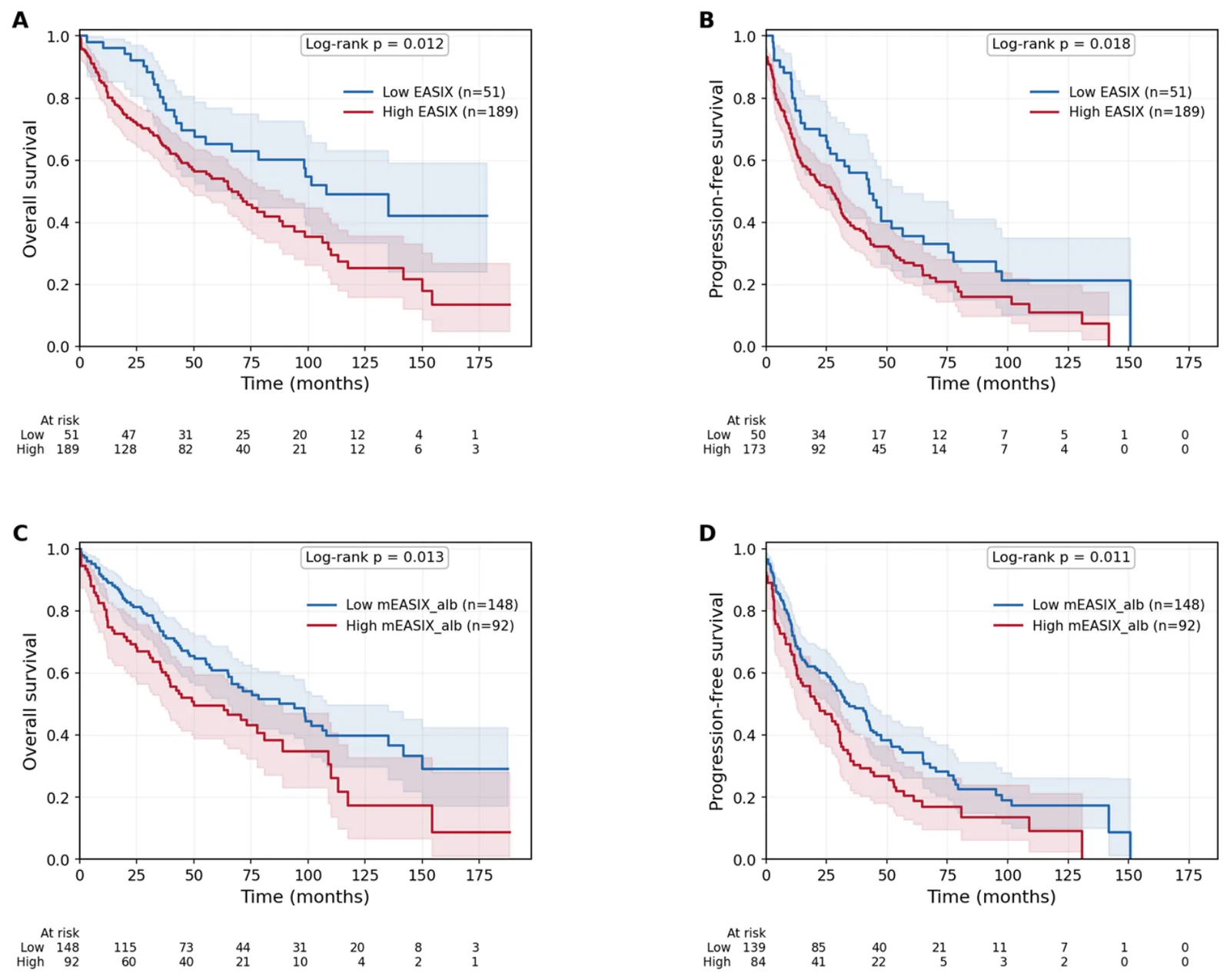
**Kaplan–Meier survival curves by EASIX and mEASIX_alb risk groups**. (**A**) Overall survival by EASIX binary risk group (cutoff > 0.471). Low EASIX (n = 51; blue) vs. High EASIX (n = 189; red). (**B**) Progression-free survival by EASIX risk group. (**C**) Overall survival by mEASIX_alb risk group (cutoff > 0.208). (**D**) Progression-free survival by mEASIX_alb risk group. Shaded areas represent 95% confidence intervals. At-risk tables are shown below each panel.

**Figure 2:**
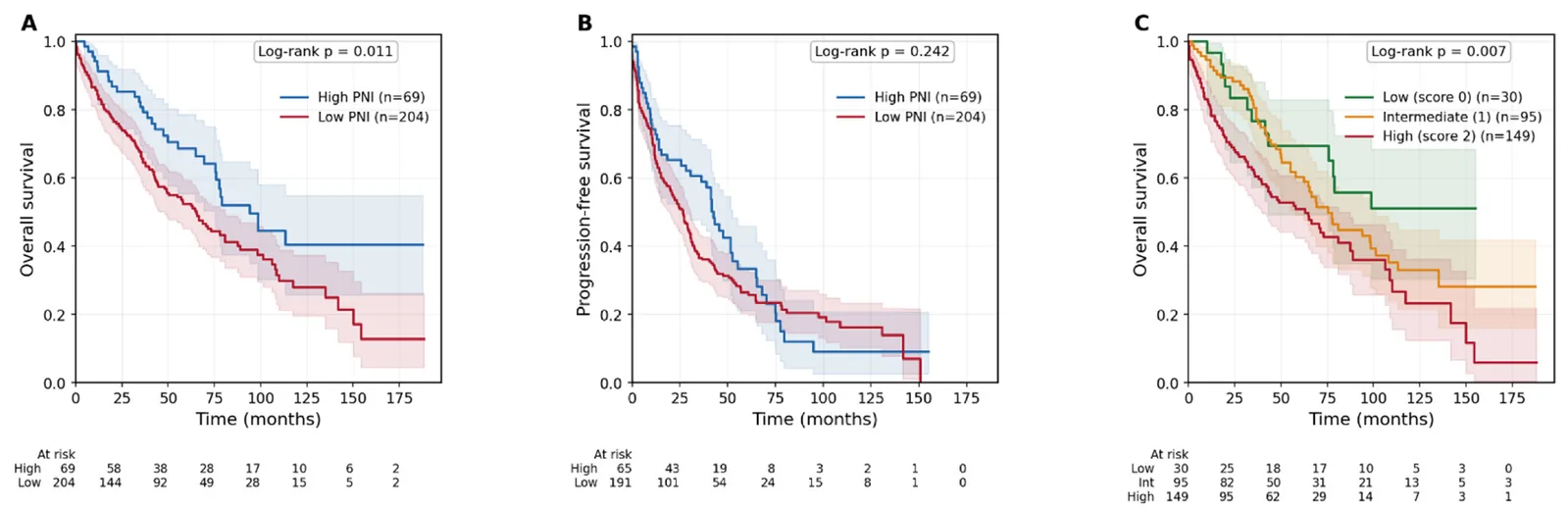
**Kaplan–Meier survival curves for PNI and Combined Risk Score**. (**A**) Overall survival by PNI risk group (PNI < 52.29 = low/unfavorable). (**B**) Progression-free survival by PNI risk group. (**C**) Overall survival by combined EASIX + PNI risk score (0 = low, 1 = intermediate, 2 = high). Log-rank *p*-values are indicated.

High EASIX (>0.471) was associated with significantly shorter OS (median 66.7 vs. 108.1 months; log-rank *p* = 0.012) and PFS (27.4 vs. 42.7 months; *p* = 0.018) (Figure 1A,B). High mEASIX_alb (>0.208) was similarly associated with worse OS (50.0 vs. 94.2 months; *p* = 0.013) and PFS (21.9 vs. 34.5 months; *p* = 0.011) (Figure 1C,D). Low PNI (<52.29) predicted shorter OS (65.2 vs. 98.6 months; *p* = 0.011) but not PFS (26.8 vs. 42.7 months; *p* = 0.242) (Figure 2A,B). mEASIX_crp did not significantly discriminate survival (OS HR = 0.999; *p* = 0.941) and was not pursued further.

### 3.4. Univariate Cox Regression Analysis

For OS, significant predictors included ISS stage (HR 1.455 per level, 95% CI 1.142–1.854; *p* = 0.002), pre-transplant response (HR 1.256, 95% CI 1.001–1.577; *p* = 0.049), age at transplant (HR 1.020, 95% CI 1.002–1.039; *p* = 0.031), EASIX (HR 1.088 per unit, 95% CI 1.047–1.131; *p* < 0.001), mEASIX_alb (HR 1.221, 95% CI 1.102–1.352; *p* < 0.001), and PNI (HR 0.973, 95% CI 0.949–0.999; *p* = 0.038). CONUT was not a significant OS predictor (HR 1.091; *p* = 0.142). For PFS, significant univariate predictors were ISS stage (HR 1.312; *p* = 0.005), pre-transplant response (HR 1.228; *p* = 0.046), lenalidomide maintenance (HR 1.676; *p* = 0.002), EASIX (HR 1.070; *p* = 0.001), and mEASIX_alb (HR 1.182, 95% CI 1.063–1.338; *p* = 0.002). PNI was not a significant PFS predictor according to univariate analysis (HR 0.983; *p* = 0.179) (Table 2). Spearman correlation confirmed an inverse relationship between EASIX and PNI (r = −0.253, *p* < 0.001).

### 3.5. Multivariable Cox Regression Analysis

OS-Model 1 (EASIX + Clinical): After adjustment for baseline covariates (ISS stage, pre-transplant response, and age), log-transformed EASIX remained an independent predictor of OS (HR 1.40, 95% CI 1.10–1.77; *p* = 0.006) and PFS (HR 1.23, 95% CI 1.01–1.50; *p* = 0.037). The estimate was essentially unchanged whether or not lenalidomide maintenance was included (OS HR 1.39 vs. 1.40), confirming that the EASIX signal does not depend on this covariate.

OS-Model 2 (mEASIX_alb + Clinical): mEASIX_alb was independently associated with OS (HR 1.38, 95% CI 1.09–1.73; *p* = 0.007), with consistent significance of all clinical covariates.

**Figure 3:**
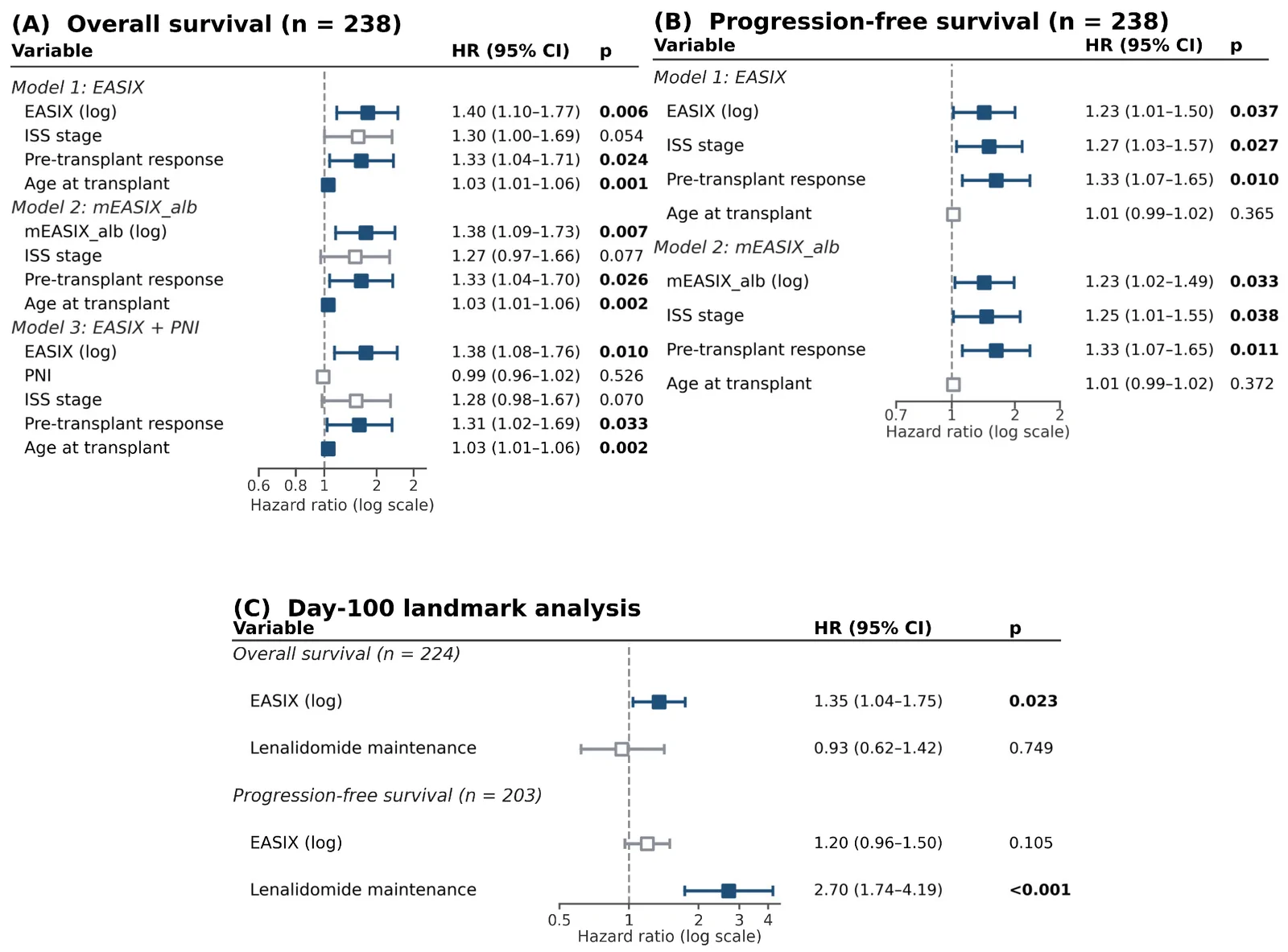
Forest plots of multivariable Cox regression results. (**A**) Overall survival and (**B**) progression-free survival multivariable Cox models, and (**C**) the day-100 landmark analysis. Each row shows the adjusted hazard ratio (square) with its 95% confidence interval (horizontal line); filled dark-blue squares indicate statistically significant associations (p < 0.05) and open grey squares indicate non-significant associations. The vertical dashed line marks HR = 1 (no effect) and the horizontal axis is on a logarithmic scale. In (C), EASIX remained an independent predictor of overall survival, whereas the maintenance associations illustrate immortal-time bias (the apparent OS benefit disappears) and confounding by indication (the adverse PFS association persists).

OS-Model 3 (EASIX + PNI + Clinical)—Key Finding: When EASIX and PNI were simultaneously included, EASIX retained prognostic significance (HR 1.38, 95% CI 1.08–1.76; *p* = 0.010), whereas PNI was non-significant (HR 0.99, 95% CI 0.96–1.02; *p* = 0.526). Notably, PNI was already non-significant after adjustment for clinical covariates alone (ISS stage, response, age; HR 0.98, 95% CI 0.96–1.01; *p* = 0.22), before EASIX was entered. The loss of PNI’s independent significance, therefore, cannot be attributed specifically to EASIX and instead reflects collinearity with clinical stage and albumin-based risk; PNI did not provide independent incremental prognostic value beyond the clinical model (Table 3, Table 4 and Table 5; Figure 3A).

PFS Models: log-transformed EASIX (HR 1.23, 95% CI 1.01–1.50; *p* = 0.037) and mEASIX_alb (HR 1.23, 95% CI 1.02–1.49; *p* = 0.033) were independently associated with PFS after adjustment for baseline covariates (Table 3, Table 4 and Table 5; Figure 3B).

**Table 3:** Multivariable Cox regression—overall survival (n = 238).

Variable	Model 1: EASIX HR (95%CI)	Model 1 *p*	Model 2: mEASIX_alb HR (95%CI)	Model 2 *p*	Model 3: EASIX + PNI HR (95%CI)	Model 3 *p*
EASIX (per ln-unit)	1.40 (1.10–1.77)	0.006	—	—	1.38 (1.08–1.76)	0.010
mEASIX_alb (per ln-unit)	—	—	1.38 (1.09–1.73)	0.007	—	—
PNI (per unit) * KEY FINDING *	—	—	—	—	0.99 (0.96–1.02)	0.526
ISS stage (per level)	1.30 (1.00–1.69)	0.054	1.27 (0.97–1.66)	0.077	1.28 (0.98–1.67)	0.070
Pre-transplant response	1.33 (1.04–1.71)	0.024	1.33 (1.04–1.70)	0.026	1.31 (1.02–1.69)	0.033
Age at transplant	1.03 (1.01–1.06)	0.001	1.03 (1.01–1.06)	0.002	1.03 (1.01–1.06)	0.002

PNI is non-significant (*p* = 0.53) once EASIX and clinical covariates are included; its attenuation is not specific to EASIX (see Results). Maintenance is omitted from the primary models and shown in Table 5.

**Table 4:** Multivariable Cox regression—progression-free survival (n = 238).

Variable	Model 1: EASIX HR (95%CI)	Model 1 *p*	Model 2: mEASIX_alb HR (95%CI)	Model 2 *p*
EASIX (per ln-unit)	1.23 (1.01–1.50)	0.037	—	—
mEASIX_alb (per ln-unit)	—	—	1.23 (1.02–1.49)	0.033
ISS stage (per level)	1.27 (1.03–1.57)	0.027	1.25 (1.01–1.55)	0.038
Pre-transplant response	1.33 (1.07–1.65)	0.010	1.33 (1.07–1.65)	0.011
Age at transplant	1.01 (0.99–1.02)	0.365	1.01 (0.99–1.02)	0.372

**Table 5:** Day-100 landmark analysis (OS n = 224; PFS n = 203). Maintenance is shown to illustrate immortal-time bias; EASIX remains robust.

Variable	OS HR (95% CI)	OS *p*	PFS HR (95% CI)	PFS *p*
EASIX (per ln-unit)	1.35 (1.04–1.75)	0.023	1.20 (0.96–1.50)	0.11
Lenalidomide maintenance	0.93 (0.62–1.42)	0.75	2.70 (1.74–4.19)	<0.001

### 3.6. Combined Easix + PNI Risk Score

The combined risk score identified three prognostic groups for OS: low-risk (score 0, n = 30; median OS not reached), intermediate-risk (score 1, n = 94; median OS 77.7 months), and high-risk (score 2, n = 150; median OS 63.3 months; log-rank *p* = 0.007). A parallel gradient was observed for PFS (45.7, 32.6, and 21.9 months, respectively; log-rank *p* = 0.115) (Figure 2C; Table 6).

### 3.7. Engraftment Kinetics

Pre-ASCT EASIX and PNI categories were not significantly associated with neutrophil or platelet engraftment kinetics. The EASIX groups showed a median neutrophil engraftment of 11 days in both groups (Wilcoxon *p* = 0.886) and platelet engraftment of 12 vs. 13 days (*p* = 0.064). The PNI groups showed no significant differences in engraftment (neutrophils, *p* = 0.596; platelets, *p* = 0.290), suggesting that the survival impact of EASIX operates through long-term mechanisms rather than acute graft kinetics.

### 3.8. Bootstrap Internal Validation

Model discrimination was assessed by Harrell’s C-index with bootstrap optimism correction (B = 1000). Optimism was minimal across all four primary Cox models (range 0.013–0.017), yielding bootstrap-corrected C-indices of 0.652 (OS, EASIX model), 0.649 (OS, mEASIX_alb model), 0.586 (PFS, EASIX model), and 0.584 (PFS, mEASIX_alb model), indicating that the continuous models’ discrimination was not inflated by overfitting. The stability of the maximally selected OS cutoff was examined separately: across 1000 bootstrap replicates, the optimal EASIX threshold varied widely (median 0.74, 95% CI 0.45–1.64). The specific value of 0.471 should therefore be regarded as an illustrative dichotomization rather than a validated clinical threshold; accordingly, all inferential analyses relied on EASIX as a continuous variable, and the binary cutoff was retained only for descriptive Kaplan–Meier illustration. The proportional-hazards assumption, assessed with scaled Schoenfeld residuals, was satisfied by EASIX in both the OS (*p* = 0.27) and PFS (*p* = 0.62) models; pre-transplant response and age showed mild violations in the PFS model (*p* = 0.01–0.03); in a sensitivity analysis stratifying on pre-transplant response and age tertiles, EASIX remained prognostic (OS HR 1.36, 95% CI 1.06–1.75, *p* = 0.017; PFS HR 1.26, 95% CI 1.02–1.55, *p* = 0.032). Lenalidomide maintenance grossly violated the assumption (*p* < 0.001), reinforcing its exclusion from the primary baseline models. In the day-100 landmark analysis, EASIX remained an independent predictor of OS (HR 1.35, 95% CI 1.04–1.75; *p* = 0.023), while its association with PFS was directionally consistent but attenuated (HR 1.20, 95% CI 0.96–1.50; *p* = 0.11).

### 3.9. Era-Independent Prognostic Value of Easix

To evaluate whether EASIX prognostic significance was confined to a specific treatment era, transplant year was examined as a binary covariate (pre-2018 [n = 158] vs. post-2018 [n = 116]; EASIX calculable in 137 and 103, respectively). When the era was added to the primary multivariable OS model, EASIX retained full independent significance (HR 1.11, 95% CI 1.06–1.15; *p* < 0.001), while the post-2018 era was independently associated with improved OS (HR 0.461, 95% CI 0.302–0.705; *p* < 0.001) and PFS (HR 0.572, 95% CI 0.415–0.789; *p* = 0.001). Era-stratified univariate analysis confirmed EASIX’s significance in both the pre-2018 (HR 1.078, 95% CI 1.035–1.123; *p* = 0.0003) and post-2018 (HR 1.232, 95% CI 1.028–1.478; *p* = 0.024) cohorts, supporting the era-independence of this biomarker.

**Sensitivity Analysis in Cytogenetically Characterized Patients.**

In the subset with available FISH data (n = 73 within the EASIX cohort), continuous EASIX remained associated with OS (HR 1.249, 95% CI 1.040–1.501; *p* = 0.018), and its effect was essentially unchanged after adjustment for high-risk cytogenetic status (HR 1.250, 95% CI 1.040–1.503; *p* = 0.017). The small number of high-risk-positive patients (n = 3) precludes definitive adjustment but argues against high-risk cytogenetics fully explaining the EASIX signal.

## 4. Discussion

This single-center retrospective study of 274 MM patients undergoing ASCT demonstrates that pre-transplant EASIX and mEASIX_alb are independent predictors of both OS and PFS after adjusting for established clinical risk factors. To our knowledge, this represents one of the largest MM-ASCT cohorts in which EASIX has been systematically evaluated as a pre-transplant biomarker.

EASIX was originally developed and validated in the allogeneic SCT setting, where endothelial injury, manifested as thrombotic microangiopathy, capillary leak syndrome, and sinusoidal obstruction syndrome, represents a major source of transplant-related morbidity and mortality [6,7]. Our findings extend this paradigm to the autologous setting, where graft-versus-host biology is absent, and survival is primarily determined by myeloma biology and response durability [15]. In this context, pre-transplant EASIX likely reflects the cumulative endothelial burden of prior therapy, disease-related endothelial activation, and the patient’s intrinsic vascular resilience. High pre-ASCT EASIX (>0.471) was associated with a 41-month reduction in median OS (66.7 vs. 108.1 months), a clinically meaningful difference.

The finding that mEASIX_alb independently predicted survival with an effect size comparable to EASIX on the log scale (multivariable HR 1.38 vs. 1.40) is biologically coherent. Albumin, incorporated into the mEASIX_alb denominator, is a negative acute-phase reactant whose depression captures systemic inflammation and nutritional depletion beyond what LDH, creatinine, and platelet count encode alone. Hypoalbuminemia is an established marker of endothelial glycocalyx degradation and increased vascular permeability [16,17]. The absence of prognostic value for mEASIX_crp (*p* = 0.941) may reflect CRP’s sensitivity to confounders in the pre-ASCT window, including recent infection and prior treatment effects.

Our analysis clarifies the relationship between EASIX and nutritional indices. PNI predicted OS in univariate analysis (HR 0.973; *p* = 0.038) but did not remain significant after adjustment for clinical covariates and provided no independent incremental value when EASIX was added (HR 0.99; *p* = 0.526). Notably, conventional EASIX (LDH, creatinine, platelets) does not contain albumin; only the albumin-modified mEASIX_alb does. The modest inverse correlation between EASIX and PNI (r = −0.253; *p* < 0.001), therefore, reflects the tendency for endothelial stress and hypoalbuminemic inflammation to co-occur in advanced disease rather than a shared analyte. EASIX, integrating three biomarkers of endothelial stress, appears to be the more informative composite. This does not diminish the clinical utility of the PNI as a simple bedside tool, but implies that when EASIX is available, standalone nutritional assessment does not provide additive OS-predictive information. The CONUT score was not independently prognostic (*p* = 0.142); myeloma biology and treatment (corticosteroids, proteasome inhibitors) can substantially alter lipid metabolism, potentially reducing the specificity of cholesterol as a nutritional marker in this population.

The combined EASIX + PNI risk score provided statistically significant OS stratification across three groups (*p* = 0.007), with the low-risk group (n = 30) not reaching median OS over an 81-month observation period. The PFS separation did not reach significance (*p* = 0.115), consistent with PNI not being an independent PFS predictor, and the high proportion in the high-risk group (54.7%) limits clinical discrimination in routine practice.

The era-independence of EASIX deserves emphasis. Our cohort spans 2009–2023, a period encompassing substantial changes in induction regimens, conditioning support, and maintenance paradigms. Despite this heterogeneity, EASIX remained a significant OS predictor in both the pre-2018 (HR = 1.078; *p* = 0.0003) and post-2018 (HR = 1.232; *p* = 0.024) subgroups, and it retained statistical significance after formal era adjustment. The post-2018 era was independently associated with improved OS (HR = 0.461; *p* < 0.001) and PFS (HR = 0.572; *p* = 0.001), likely reflecting advances in supportive care and maintenance strategies, but this did not confound the EASIX signal. Bootstrap internal validation (B = 1000) yielded corrected C-indices of 0.652 (OS) and 0.586 (PFS) with minimal optimism (0.013–0.017), confirming that the continuous model’s discrimination was not inflated by overfitting. The binary cutoff itself, however, was unstable on resampling (median 0.74, 95% CI 0.45–1.64); we therefore based all inference on continuous EASIX and present the 0.471 threshold for descriptive purposes only.

A note on the lenalidomide maintenance finding is warranted. In an exploratory time-fixed model, maintenance appeared to be associated with improved OS (HR 0.675; *p* = 0.039) yet paradoxically with shorter PFS in multivariable analysis (HR 1.47; *p* = 0.029). Two non-exclusive mechanisms likely operate. First, there is confounding by indication: maintenance was prescribed preferentially in higher-risk patients and in those transplanted more recently, who have shorter follow-up and closer progression surveillance. Second, and importantly, because maintenance begins only after transplant, patients must remain progression-free long enough to receive it; modeling it as a baseline covariate therefore introduces immortal-time bias, which tends to inflate the apparent OS benefit and is not corrected in the present analysis. These maintenance associations should thus be interpreted as hypothesis-generating rather than causal. Because maintenance is a post-transplant exposure, it was excluded from the primary models and evaluated in a day-100 landmark analysis. In that analysis, the apparent OS benefit disappeared (HR 0.93, 95% CI 0.62–1.42; *p* = 0.75), consistent with the earlier estimate being largely an immortal-time artifact, whereas the adverse PFS association persisted (HR 2.70, 95% CI 1.74–4.19; *p* < 0.001). This persistence is consistent with, but does not prove, residual confounding by indication. Because maintenance timing was not recorded, residual immortal-time bias for the maintenance term cannot be fully excluded. EASIX itself remained an independent predictor of OS within the landmark cohort (HR 1.35; *p* = 0.023). Maintenance associations should therefore be interpreted as non-causal.

Engraftment kinetics were not significantly associated with EASIX or PNI risk categories. The marginal trend for later platelet engraftment in high-EASIX patients (13 vs. 12 days; *p* = 0.064) is consistent with subclinical endothelial dysfunction affecting marrow niche recovery, but did not reach significance. This finding suggests that the survival impact of pre-ASCT EASIX operates through mechanisms beyond the acute peri-engraftment period.

Strengths include a large, well-characterized cohort with complete albumin data (100%), long follow-up (median 81.3 months), and a prespecified multivariable modeling strategy. Limitations include: the retrospective single-center design; the absence of high-risk cytogenetic (FISH) data in 68% of patients (among 88 patients with complete FISH data, only 3 harbored high-risk cytogenetics [del(17p), t(4;14), or t(14;16)], reflecting selective historical FISH utilization and precluding a formally powered subgroup analysis; nonetheless, in a sensitivity analysis restricted to FISH-tested patients, the EASIX–OS association persisted and was robust to adjustment for high-risk cytogenetics (see Results)); EASIX non-calculability in 12.4% due to missing LDH; and the lack of serial EASIX measurements at post-transplant timepoints. Although bootstrap internal validation demonstrated minimal optimism (0.013–0.017), external validation in an independent cohort is required before clinical adoption. Future prospective multicenter studies incorporating serial EASIX assessments, MRD monitorin, and complete cytogenetic profiling are warranted.

## 5. Conclusions

In this single-center cohort of multiple myeloma patients undergoing autologous stem cell transplantation, the pre-transplant EASIX and its albumin-modified variant (mEASIX_alb) were independent predictors of both overall and progression-free survival, retaining prognostic value after adjustment for ISS stage, pre-transplant response, age, and maintenance therapy. When analyzed jointly, the PNI did not provide independent incremental prognostic value beyond EASIX and established clinical covariates. The association was stable across treatment eras and was supported by bootstrap internal validation; however, the data-derived binary cutoff was less stable than the continuous index and should be regarded as illustrative rather than a definitive clinical threshold. Derived entirely from routinely available laboratory parameters, EASIX offers a simple, low-cost tool that may refine pre-transplant risk assessment. Prospective and external multicenter validation is warranted before routine clinical implementation.

## Figures and Tables

**Table 1:** Patient characteristics and pre-ASCT laboratory values (n = 274).

Characteristic	Value
Demographics	
Age at ASCT, years—median (IQR)	57.8 (51.4–63.2)
Female sex—n (%)	149 (54.4%)
Myeloma subtype	
IgG	151 (55.1%)
IgA	47 (17.2%)
Light chain	60 (21.9%)
IgM/Other	16 (5.8%)
Missing	2 (0.7%)
ISS stage	
Stage I	42 (15.4%)
Stage II	89 (32.6%)
Stage III	141 (51.5%)
Missing	2 (0.7%)
Pre-transplant response	
Complete response (CR)	156 (57.1%)
Very good partial response (VGPR)	84 (30.7%)
Partial response (PR)	33 (12.1%)
Missing	1 (0.4%)
Induction therapy	
Bortezomib + Cyclophosphamide + Dexamethasone (VCD)	187 (68.2%)
Bortezomib + Dexamethasone (VD)	61 (22.3%)
Other/Multiple regimens	26 (9.5%)
Conditioning regimen	
Melphalan 200 mg/m^2^ (MEL200)	254 (92.7%)
Reduced intensity	19 (6.9%)
Missing	1 (0.4%)
Lenalidomide maintenance	
Yes	184 (67.2%)
No	89 (32.5%)
Missing	1 (0.4%)
Engraftment	
Neutrophil, days—median (IQR)	11 (11–12)
Platelet, days—median (IQR)	13 (11–15)
Pre-ASCT laboratory (day 3)	
LDH (U/L)—median (IQR)	191 (160.5–227)
Creatinine (mg/dL)—median (IQR)	0.77 (0.67–1.00)
Platelets (×10^9^/L)—median (IQR)	228 (189–278)
Albumin (g/dL)—median (n = 274, complete)	4.1
Composite Scores	
EASIX—median (IQR); n available	0.699 (0.490–1.049); n = 240
mEASIX_alb—median (IQR); n available	0.176 (0.119–0.262); n = 240
PNI—median (IQR); n available	49.0 (45.5–52.3); n = 273
Outcomes	
Median follow-up (reverse KM), months	81.3
Deaths—n (%)	150 (54.7%)
Median OS (months; 95% CI)	71.5 (62.5–89.0)
PFS events—n (%)	207 (75.5%)
Median PFS (months; 95% CI)	29.0 (23.9–33.9)

Abbreviations: ASCT, autologous stem cell transplantation; IQR, interquartile range; ISS, International Staging System; LDH, lactate dehydrogenase; EASIX, Endothelial Activation and Stress Index; mEASIX_alb, albumin-modified EASIX; PNI, Prognostic Nutritional Index; KM, Kaplan–Meier; OS, overall survival; PFS, progression-free survival.

**Table 2:** Univariate Cox regression for overall survival and progression-free survival.

Variable	OS HR (95% CI)	OS *p*	PFS HR (95% CI)	PFS *p*
Age at transplant (per year)	1.020 (1.002–1.039)	0.031	1.002 (0.988–1.015)	0.824
Male sex	0.870 (0.630–1.201)	0.398	0.919 (0.698–1.211)	0.548
ISS stage (per level)	1.455 (1.142–1.854)	0.002	1.312 (1.084–1.589)	0.005
Pre-transplant response (per level)	1.256 (1.001–1.577)	0.049	1.228 (1.004–1.502)	0.046
Conditioning (MEL200 vs. other)	0.862 (0.438–1.694)	0.666	0.638 (0.356–1.144)	0.131
Lenalidomide maintenance	0.833 (0.591–1.173)	0.296	1.676 (1.215–2.311)	0.002
EASIX (continuous)	1.088 (1.047–1.131)	<0.001	1.070 (1.026–1.115)	0.001
mEASIX_alb (continuous)	1.221 (1.102–1.352)	<0.001	1.182	0.002
PNI (continuous)	0.973 (0.949–0.999)	0.038	0.983	0.179
CONUT (continuous)	1.091 (0.971–1.226)	0.142	-	-

EASIX and mEASIX_alb were analyzed in n = 240; PNI/CONUT in n = 273. HR, hazard ratio; CI, confidence interval.

**Table 6:** Combined EASIX + PNI risk score.

Risk Group	n (%)	Median OS, Months (95% CI)	Median PFS, Months (95% CI)
Low (score 0: EASIX low + PNI high)	30 (10.9%)	Not reached (75.9–NR)	45.7 (25.6–77.5)
Intermediate (score 1)	94 (34.3%)	77.7 (62.5–108.1)	32.6 (26.5–47.7)
High (score 2: EASIX high + PNI low)	150 (54.7%)	63.3 (42.2–87.5)	21.9 (14.4–30.7)
Log-rank *p*		0.007	0.115

Score = EASIX > 0.471 (1 point) + PNI < 52.29 (1 point). NR, not reached.

## Data Availability

The datasets generated and analyzed in the current study are not publicly available due to institutional privacy policies and participant confidentiality agreements. However, de-identified data may be made available by the Corresponding Author upon reasonable request, provided appropriate ethical approvals are in place. The data presented in this study are available on request from the corresponding author due to ethical restrictions.
